# Exploring neurodevelopmental outcome measures used in children with cerebral malaria: the perspectives of caregivers and health workers in Malawi

**DOI:** 10.1186/s12887-016-0763-y

**Published:** 2017-01-10

**Authors:** Emmie W. Mbale, Terrie Taylor, Bernard Brabin, Macpherson Mallewa, Melissa Gladstone

**Affiliations:** 1 Malawi-Liverpool Wellcome Trust Clinical Research Programme, PO Box 30096, Chichiri, Blantyre, 3 Malawi; 2 Paediatric department, University of Malawi, College of Medicine, P/Bag 360, Blantyre, 3 Malawi; 3 College of Osteopathic Medicine, Michigan State University, East Lansing, MI USA; 4 Clinical Sciences Division, Liverpool School of Tropical Medicine, Pembroke Place, Liverpool, UK; 5 Department of Women and Children’s Health, Institute of Translational Medicine, University of Liverpool, Alder Hey NHS Children’s Foundation Trust, Eaton Road, Liverpool, L12 2AP UK; 6 Blantyre Malaria Project, University of Malawi College of Medicine, Blantyre, Malawi

**Keywords:** Cerebral malaria, Neurodevelopment, Core outcomes, Clinical outcomes, Neurodisability, Africa, Qualitative, Interviews, Family perspectives, Child disability

## Abstract

**Background:**

Progress has been made in tackling malaria however there are still over 207 million cases worldwide, the majority in children. As survival rates improve, numbers of children with long-term neurodisabling sequelae are likely to increase. Most outcome studies in cerebral malaria (CM) have focused only on body function and structure and less on outcomes within the broader framework of the International Classification of Functioning and Disability (ICF). The aim of this study was to utilise qualitative methods to identify relevant clinical outcomes in CM to support formulation of a core outcome set relevant to CM and other acquired brain injuries for use in future clinical trials.

**Methods:**

In depth interviews with parent/caregivers (CGs) of children with/without previous CM (*N* = 19), and in depth interviews with health professionals (*N* = 18) involved in their care were conducted in community and clinical settings in and around Blantyre, Malawi. Interviews were audio taped, transcribed, translated and a thematic content analysis was conducted. Themes were categorised and placed firstly in an iterative framework derived from the data but then within the ICF framework.

**Results:**

Outcomes perceived as important to carers and professionals fulfilled each level of the ICF. These included impairment in body function and structure (contractures, impaired mobility, visual problems, seizures, cognitive function and feeding); activity and participation outcomes (learning, self-care, relationships in school, play and activities of daily living). Other issues emerging included the social and emotional implications of CM on the family, and balancing care of children with neurodisability with demands of daily life, financial pressures, and child protection. Themes of stigma and discrimination were described; these were perceived to negatively influence care, participation and integration of carer and child into the community.

**Conclusions:**

Outcomes considered important for parents/caregivers and professionals working with children post CM cross all aspects of the ICF framework (impairment, functioning and participation). Outcomes emphasised by families and carers in cross-cultural settings must be given adequate attention when conducting clinical studies in these settings.

**Electronic supplementary material:**

The online version of this article (doi:10.1186/s12887-016-0763-y) contains supplementary material, which is available to authorized users.

## Background

Cerebral malaria (CM) is a serious complication of malaria. It is characterized by peripheral *Plasmodium falciparum* parasitaemia and profound coma without an identifiable alternative cause [1, 2]. Mortality rates reach 20%, even with the best care. Outcome studies to date have demonstrated that of those who survive, approximately 35% have some form of neurodisability (impairment in neurological function) or neurodevelopmental disorder [3–6]. Tools to assess children for these studies have concentrated mainly on specific impairments (spasticity, hemiplegia, quadriplegia) [7], cognitive function [8] or behaviour [9], and most have used Western outcome measures translated and in some cases, adapted for an African setting. A recent literature review emphasized how very few studies have taken a holistic approach to outcomes and how most [10] report effects on body and function but not on participation. Furthermore, many studies report the general domains affected, but provide little information on the assessment processes used [11–13]. Other potentially useful information, such as school attendance, socioeconomic status and maternal education, all of which can impact on a child’s neurodevelopmental outcomes have often not been considered.

The World Health Organisation (WHO) defines disability as “an umbrella term, covering impairments, activity limitations, and participation restrictions” [14] combining both the medical model and the social aspects of disability. Within this context, the International Classification for Functioning, Disability and Health (ICF) has been designed to act as a contextual framework for the reporting the outcomes of disability. This can be used both in health care and research, and as a tool for planning and monitoring interventions [15]. It has two components separated into four main areas; a) body parts/structure and body function, b) functioning and participation of the individual in their environment c) contextual factors including environmental influences and d) individual differences [15].

As yet there is no agreed set of outcomes to clearly compare and measure the impact of interventions for CM or for many neuro-infectious or neuro-disabling conditions. This poses difficulties when comparing the impact of interventions, and interferes with the ability to summarize comparative evidence on clinical outcomes [16].

Core outcome sets (COS) have recently been presented as a way of having “an agreed standardized collection of outcomes that should be measured and reported in all trials for a specific clinical area” [17]. COS are considered relevant in any clinical trial, and any set created should meaningfully encompass a consensus view. It is expected that patients and their parents/caregivers will be involved in the development of these sets of core outcomes, in order to ensure that the research process is more relevant and appropriate to patient’s needs. Patient and CG input can add a unique dimension as they draw from experiences, knowledge and perspectives that may be missed by researchers and health professionals [17, 18]. In asthma, child and parental involvement in COS development resulted in emphasis on long-term treatment effects, [19], and in rheumatology, symptoms related to the quality of life have been subsequently included as outcomes for affected patients [20].

As the first step in creating a COS for CM and other neuro-infectious diseases in children in an African setting, this study aimed to use qualitative methods to understand the concepts, themes and views of Parent/caregiver and health professionals in relation to outcomes in children post cerebral malaria.

## Method

### Type of study

Qualitative research methods were used to explore perceptions and meanings attached to issues through the “participant’s eyes” [21–23]. We hypothesised that parents/caregivers and health professionals would have their own “realities, shaped by their experiences, culture and background”, and that these would define their priorities for children with CM [24].

### Study setting

This study was conducted in Queen Elizabeth Central Hospital in both hospital and outpatient settings (physiotherapy and paediatric neurology clinics) as well as one main community neurodisability centre in central Blantyre.

### Sampling

Purposive sampling methods were used in which participant selection was based on the participant’s experiences and characteristics [25]. We chose to select home-based parents/caregivers who were either urban/rural dwellers; parents of school going or of pre-school children, and parents of those with and without disability. This sampling enabled a mixture of socio-demographic and health features and included those with more or less severe sequelae post CM [26]. Nineteen parents/caregivers were recruited (5 males and 16 females, 8 from rural settings and 11 from urban settings) who either had children with confirmed CM [27, 28] or who had children who had had CM and who were now attending paediatric neurology and physiotherapy clinics, or who were seen within community groups for carers (Table 1).

**Table 1:** Table of carer participant characteristics (*N* = 19)

Participant code	Age range of participant’s child (years)	Post CM sequelae if CM in participants child
CG1	6–10	Cerebral palsy
CG2	0–5	No post CM neurodisability
CG3	6–10	No post CM neurodisability
CG4	6–10	Cerebral palsy
CG5	11–15	Behavioural and learning difficulties
CG6	0–5	Behavioural difficulties/ developmental delay
CG7	0–5	No post CM neurodisability
CG8	0–5	No post CM neurodisability
CG9	0–5	No post CM neurodisability
CG10	0–5	Developmental delay
CG11	0–5	No post CM neurodisability
CG12	6–10	No post CM neurodisability
CG13	6–10	Epilepsy
CG14	11–15	Cerebral palsy
CG15	11–15	Hemiplegia
CG16	11–15	Behaviour and learning difficulties
CG17	6–10	Behaviour and learning difficulties
CG18	11–15	Behavioural difficulties and hemiplegia
CG19	0–5	No post CM neurodisability

Eighteen nurses, clinicians, and other health workers who were involved in the children’s rehabilitation and palliative care were interviewed to obtain their views on different aspects of care. Clinicians who provided care during the acute care phase, and who were responsible for follow-up were included. These comprised nurses providing in-patient support and follow-up care including neurodevelopmental assessment, physiotherapists, palliative care nurses, and clinicians who provided support for patients with chronic conditions both in hospital and at home (Table 2).

**Table 2:** Table of professional participant characteristics

Characteristics	Number interviewed
Nurses:	
Provide mainly nursing care during admission and follow-up and responsible for conducting neurodevelopmental assessments.	6
Clinicians:	
Clinical officers, medical doctors, paediatricians involved in acute and follow-up care.	6
Other health professionals	
Rehabilitation experts (physiotherapists, occupational therapists, palliative care workers).	6
Total	18

### Data collection

Interviews took place in Blantyre Malawi within a quiet facility either at the tertiary paediatric teaching hospital, (Queen Elizabeth Central Hospital), or within a community group setting for parents/caregivers of children with neurodisabilities in an urban township. Demographic data on parents/caregivers and the children they cared for was obtained. This included age, sex, level of education, and relationship of the CG to the child. The interviewers conducted in depth interviews which allowed for a detailed understanding of the participants’ views and experiences in their own words, and provided the space for them to raise the issues they considered important to them and/or their families [24]. In depth interviews enabled families to reveal information that may not be easily raised in an open forum such as a focus group discussion. Within the in depth interviews, a narrative approach was used to enable information regarding the totality of the experience with CM to be gathered in an open manner. This enabled a better understanding of outcomes expected, and whether those were achieved.

Interviews and transcribing were completed by a medical doctor who manages children with neurological disorders (EM) and a social scientist (MC), both have had experience and training in qualitative research methods. Findings were discussed within the research team and the coding framework was developed jointly.

Interviews were conducted in English or Chichewa, depending on the participant’s preference and to improve rapport. All interviews were audio recorded, using notes to keep track of questions, maintain focus, and to record non-verbal communication [29]. Topic guides were used to guide the discussion and were flexible in order to allow topics to be explored during the flow of the discussion (see Additional files 1 and 2). Using an inductive approach, we chose not to provide participants with a suggested list of outcomes as has been done in other studies [17], as that could limit or restrict participants to focussing on those suggested outcomes. A topic guide was used with open-ended questions to facilitate this.

Each interview helped to inform and shape the topic guide by identifying areas needing further probing and clarification. All interviews were transcribed and translated from Chichewa into English. Using Chichewa as the primary language for the study enables the language emerging from the study to be used in any outcome measures created or adapted for use in Malawi in the future. The principle of saturation was used with data collection until no new information emerged [24]. Nineteen interviews were conducted with withparent/caregivers and 18 interviews were conducted with health professionals. We used an iterative process to determine saturation particularly when construct domains were complete.

### Consent procedures for all participants

All potential participants received structured information about the purpose and procedures of the study, in written form (participant information sheet) but also explained through the translator as many carers could not read. A written informed consent form was then completed and verbal consent was recorded by Dictaphone before commencing any work. All transcripts were given a code which was stored in a password protected file to which only the researchers had access.

### Data management and analysis

Analysis involved transcript familiarisation, identifying themes emerging from the data and manually developing a coding framework [30, 31]. Coding and retrieval of data was supported by NVIVO 10.0 which enhanced accountability of the data. Emerging themes were compared between different groups and related back to the study objectives and content analysis [24]. Segments of text relevant to each theme were extracted to maintain the essence and meaning of the text. Data were coded into the main themes and sub-themes of the World Health Organisation International Classification of Functioning and Disability.

## Results

In total, 37 interviews were undertaken. Carers of 19 children, both preschool (≤6 years) and school aged (≥6 years) were interviewed. Eight were from more rural areas of Blantyre district and 11 from heavily urbanised settings within Blantyre City. The parents or caregiver’s education ranged from standard 4 (primary only) to form 3 (started but not completed secondary education), and all were female. 11 parent/caregivers had children with disabilities and 8 had children without known disabilities (Table 1). The 18 health professionals interviewed comprised clinicians (two clinical officers, 4 paediatricians); nurses; rehabilitation technicians and palliative care workers. (Table 2)

**Fig. 1:**
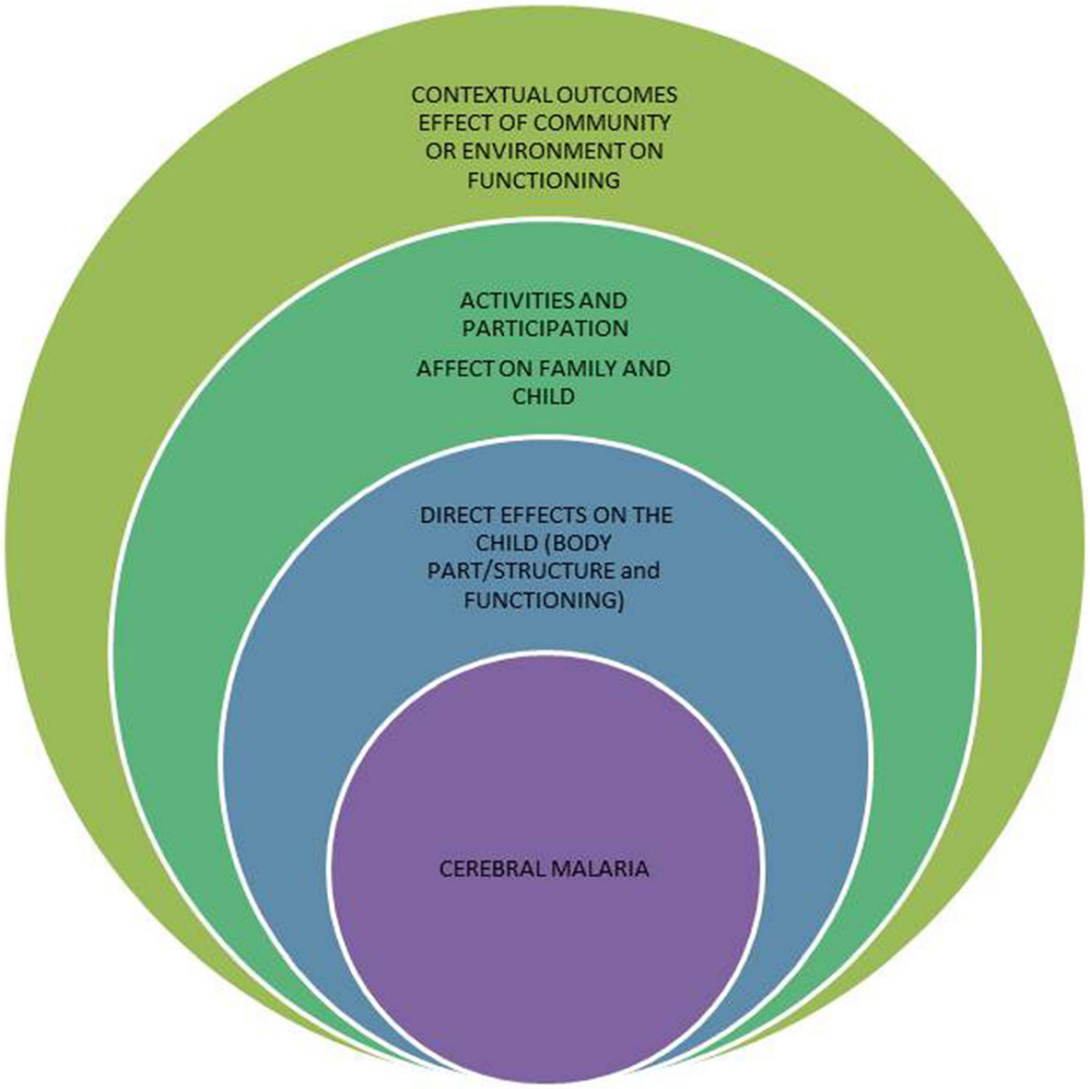
Categorisation of outcomes in cerebral malaria according to the International Classification of Functioning and Disability

Participants described outcomes of CM in relation to the health of the children, the social implications on the child; the impact of CM on the family unit, and the social context in which this takes place (Fig. 1).

Results are presented within the framework shown in Fig. 1. The categories follow the ICF framework and are divided into A) Direct effects of CM on the child (body parts/structure and functioning), B) Activities and Participation and effects of CM on activities and functioning for the child and family, and C) Contextual outcomes and effects of CMon the community and environment. Example quotes for each category are placed within Tables 3, 4 and 5 according to the three areas of the ICF framework, as well as quotes from parents/caregivers and professionals.

**Table 3:** Quotes of participants on outcomes of CM relating to Body Function and Structure

Functioning and Disability		
*Body Function*	Carers views	Professional views
Mental Function	*“the head doesn’t function normally mutu suugwila nchito bwino” (CG 2)*	
Sensory Function		
*Hearing Function*	“*now I can differentiate because she was not hearing but now she can hear.” (CG 8)*	*“Mother said when I was being discharged I was told will have hearing problem and indeed when we were trying to communicate could not hear but slowly has started picking up. (Nurse 1)*
*Seeing Function*	*“My concerns are on school since she can’t see properly so my worry is could she continue with school with no problems” (CG 12)*	*“Whilst they are still in the hospital here they lose their sight which of course most of them do regain it” Nurse 3* *“That’s what we feel, eh, the child says”I was able to see but now I’m not seeing” so you develop some behavioural problems” (Rehabilitation professional)*
Movement functions		
*Involuntary movement functions (seizures)*	*““I say “how can I travel, what if he convulses on the way, what will I do?” (CG 2)* *“Now he just blinks continually but before he would have frank seizures” (CG 4)*	*“Same thing with epilepsy we might not see it in the acute phase but a year down the line post malaria episode you might start to see epilepsy developing”. (Clinician 1)*
*Control of voluntary movement functions*	*“the hand was fist closed could not open and the leg was not in its normal position” (CG 1)* *“Her arm….she is unable to lift things” (CG 2)*	*“I was thinking on motor should be emphasised since it is supporting the others since when the gross motor is not good fine motor is not good it also affects the cognitive”. (Nurse 2)* *“Then there are other children who lose their sight some get paralysed one side or a certain body part is not working properly..” (Nurse 3* *“The complications parents will be worried because from that point they will have regression in neuro development” (Clinician 1)*

**Table 4:** Quotes of participants relating to Activities and Participation of children post cerebral malaria

Activities and Participation		
	Carer’s views	Professional views
Learning and Applying Knowledge		
*Basic Learning Acquiring skills*	*At that time (before CM) he was more alert/clever (anali wochangamuka). At the moment maybe…it looks like he is going backwards/regressing (kubwelela mmbuyo)” (CG 10)* *“Before he got sick things were ok he was intelligent, was first in his class” (CG 12)*	*“No most of them do not raise performance as a problem but when we do the assessment we note that the child cannot even perform in school” (Nurse 1)*
*Applying knowledge Focusing attention*	*follow instructions; “When you send her, she comes back with something else, she keeps forgetting” (CG 8)* *“the difference is that when the other was younger, you could see that her head is working ok (mmutumu zimayenda bwino bwino). Because if you send her she would do what you say and when she went to play, she wouldn’t undress.”* (CG 3)	*“They become some sort of dull they are not concentrating when the child is going to school” (Nurse 4)* *“Cognitive problems, psychological issues, things like behaviour aggressive behaviour, because that yes is a major issue that can disrupt the whole family and has a major consequences in terms of school, schooling for the kid” (Clinician 2)*
General Tasks and Demands		
*Managing one’s own behaviour*		*“the child may appear to be completely well but his/her behavior is still a problem. He is very destructive, keeps beating his/her friends” (Nurse 3)“* *“The child may be school aged but cannot go to school because he is very destructive. The other children would be in trouble and even his child’s life is not safe because if the child meets some people who are unaware of his/her problems and he/she provokes them, they may hurt him/her and so such a destructive child needs proper assessment.”* (Nurse 5)
Communication		
		*“On speech there are some patients that are stroke patients even the children with CP they also have the same problems” (Rehab technician)*
Mobility		
*Walking*	*“Her legs became paralyzed…she was able to walk before the illness and when she suffered from malaria she stopped walking”* (CG 1) “*We stayed for almost a week at home and then she started walking again*” (CG 8)	*“they’re not able to support themselves, they are unable most of the times to walk”(palliative care professional)*
*Moving Around*	*“He is going backwards (kubwelela m’mbuyo)…he was crawling at that time (pre CM) but now he is not crawling”* (CG 3)	
Self Care		
*Feeding*	*“For instance with eating nsima, he coughs, struggling with swallowing but when we give I porridge it is better” (CG 2)*	*“So this causes a challenge because quite often they are not feed themselves (Palliative care professional)* *“The child has been discharged on nasal gastric tube because of …the brain injury that he cannot even swallow” (Nurse 2)* *“When you arrive at the house and hear the breathing and you realize that the chest is congested because of the feeding practice. The big problem is the method of feeding, so you show them the correct position for feeding because if the child is being fed whilst lying down, the child is at risk of choking and also of pneumonia.” (Palliative care nurse)*
Domestic Life		
*Preparing meals*	*but she is able to put cold water on fire she holds with one arm and also is able to set fire.(CG 15)*	*“..most of the times they are perceived as time wasters for the mothers..instead of maybe mothers concentrating on some of the households chores, attending to the siblings attending to the husband, themselves they need much time to attend to these children” (nurse 5)*
Interpersonal Interactions		
*General interpersonal interactions*	*“Iiiih! [emphasis] As of now she is really changed, stealing! Whatever she finds in the house whether a friend has left nsima on the table she just grabs it.” (CG 1)*	*“but there are a few will complain that my child after the malaria became much more aggressive than before they are always fighting us through (Clinician 5)*
*Basic interpersonal interactions*		“*After…. CM they were behaving like those children with mental disorders. Most of the time they can hold any doctor when he comes they can hold this and take that they cannot really respond to what mother or nurses are doing”.* (Nurse 6)
*Particular interpersonal relationships*	*“It is not easy to help another man’s child. It is not easy.“ (CG 16)*	
*Informal social relationships*		*“the main thing is that they lack knowledge, if they knew that taking the child outside and allowing him to be and play with other children could help his brain, maybe, they may do this more.” (Palliative care professional)*
Major Life Areas		
*Education*	*“So my worry is could she continue with school with no problems” (CG 9)*	
*Work and employment*		*“When they are coming for the next visit other children improve depending on what the mother does at home but with Malawian socio-statuses other children remain delayed when the mother is a busy woman”. (Nurse 2)*
*Economic life*	*“So we try to get the food he eats, its the kind that we could not afford during that time but we will settle for ‘thelele’ (wild vegetable) so that our child can eat the things that we feel he is able to eat happily.” (CG 7)*	*“If the child is like in this status, it means the family has to source out the money to travel to access the Occupational therapy” (Rehabiliation professional)*
Community Social and civic life		
*Community life*		*“…they cannot perform the activities within their communities because there is this child who is like now incapacitated, so they need to be close to him. They cannot participate in any other church activities because they must look after this child in each and every moment so it is like the holistically they are affected in all aspects” Rehab/palliative care nurse*
*Religion and Spirituality*	*“the discussion we had was that may be he has been bewitched and we should just seek traditional help like at the village the way they know epilepsy like convulsing” (CG 1)* *“why are you just looking at the child like that without consulting a traditional healer or going for prayers?” (CG 15)*	“They think this has just happened because of people (witchcraft)” (Nurse 4)

**Table 5:** Quotes of participants relating to contextual factors and their effect on outcomes in children post cerebral malaria

Contextual Factors		
	Carer’s views	Professional views
Support and relationships		
*Friends (of child or parents)*	*“So when I am away maybe to the market. When she wants to respond to nature’s call it’s her friends who carry her. But they are growing and they also need to go to school.”* (CG 16)	“They’re not able to support themselves, they are unable most of the times to walk and become completely dependent to the people who are around them. So for their quality of life to be achieved, it is dependent of the people who surround them”. (Palliative care professional)
Attitudes		
*Individual attitudes of Immediate and extended family*	*“This is a very big challenge (having a child with neurodisability) which you cannot manage to handle on your own, you need a relative to help you” (CG 2)*	*“become completely dependent to the people who are around them. So for their quality of life to be achieved, it is dependent of the people who surround them” (nurse 5)*
*Societal attitudes*	*“My social life is really affected, when I go out, my neighbors shun me (amandisala)… Im just alone in my house with my child… When I go outside, I am treated with reproach/ shame (chimakhala ndithu chitonzo)” (CG 4)*	

The theme of neurological impairment was common, and there were many quotes from all interviewed groups. However, frequent issues included those relating to behavioural problems of children with cerebral malaria, the financial implications for families of a child with CMand general issues concerning community child participation at school and at home. These are described within the subsections below which are broken up into the ICF framework.

### A. Direct effects of CM on the child (body part/structure and functioning)

#### Survival

Survival of the child was, not surprisingly, mentioned as being the outcome of primary importance to both health professionals and parents/caregivers. *“They (health professionals) really helped me because when I was taking him home, he was fine, he was alive” (CG 3).* One clinician cited that in view “*of the turbulent course of CM”* and the high mortality, survival was *“fantastic!!”* Others echoed these sentiments; *“…the child comes in with cerebral malaria, they are comatosed, they are convulsing and…you are caring for them you feel like some of them will succumb to death but when they recover even though they recover with a sequelae you feel like you have done something to rescue this child” (Clinician 1).*

### Body functions

#### Sensory function

Sensory functions, such as hearing and visual loss, were discussed rarely by professionals as outcomes that in some cases improved with time, but if not, were a source of distress to parents/caregivers. *“My concerns are on school since she can’t see properly so my worry is ‘could she continue with school with no problems?” (CG12). Professionals also described this as being a common problem post CM; “The mother said when she was being discharged she was told will have hearing problem and indeed when we were trying to communicate…. could not hear but slowly has started picking up” (Nurse 5).*

#### Change in motor function (voluntary motor function or involuntary)

Direct neurological damage/consequences emerged as an area of common concern to all participants. Descriptions of motor disorders included: “not walking”, “weak limbs”, “upper motor neuron”, developmental regression and movement disorders. Regaining motor function was a target for rehabilitation teams, clinicians and nurses particularly as a CM survivor was only considered a “normal person” by some parents/caregivers if they could move themselves; *“At that point he had been walking, talking doing everything normally like a normal person/human being” (zonse bwino bwino ngati munthu)” (CG4). “She was able to walk before the attack and when she suffered from malaria she stopped walking even by the time we were being discharged she was not walking. We stayed for almost a week at home and she started walking again”* (*CG15).*

*Some parent/caregivers described how vital normal movements were for children and people generally; “Because as a human being/normal person (munthu) you need to turn yourself, stretch your arms, speak, that’s when you know that things are well” (CG7).*

For those families of children with cerebral malaria, not all children had neurodisability post CM. Some parents reported children being the same after the illness; *“Right now she is doing what she was doing when she was ok and now there is no difference” (CG15).*

#### Epilepsy or seizure disorders

Seizures were perceived by clinicians as being a common outcome in CM; *”mostly its patients who come in with new onset seizures after a battle of cerebral malaria” (Clinician 1); “She startles frequently, when I sit her down she keeps startling” (amadzidzimuka pafupipafupi) (CG12).* Although parent/caregiver described children having various types of seizures, surprisingly they never actually mentioned the Chichewa word for epilepsy “khunyu”, a word that relates to episodes of collapse and fits thought to be as a result of evil spirits. Many of our families knew that their child had an underlying cause for their seizures such as CM and therefore would not use this word. Furthermore, chunky is a terminology related to those having a tonic-clonic convulsion with foaming at the mouth and incontinence, so other seizures (focal motor, complex partial) would not manifest like this.

### B. Activities and participation

A high proportion of emerging themes related not only to direct effects of CM on the child but also to effects on the child and family functioning, activities and participation.

#### Learning and applying knowledge

Only health professionals used the term “cognitive problems”, but various aspects of cognition, memory loss, learning difficulties and academic performance were described by parents/caregivers, and both groups indicated this was an important outcome for families and children.

#### Acquiring skills

Forgetfulness was described in the context of a child’s ability to follow instructions; *“When you send her, she comes back with something else, she keeps forgetting and also slowness on doing things……… up to now that problem is still there” (CG8).* Professionals described it as a memory problem; *“Some lost memory, I remember only two cases that they had loss in memory like when I send a child to this s/he comes with that, the mother brought a child here to complain” (Nurse 1).* Several health professionals stated that it was necessary to specifically ask parents/caregivers about academic problems because the parents or caregivers did not volunteer academic problems as a concern. The parents or caregivers placed emphasis on academic abilities and some perceived that a child’s intellectual capacity was impaired after the illness; *“My main concern is that she does not understand properly. Even at school, it’s like she does not do well. I talked to the teacher to be placing her in front since she does not perform well. So she repeats the same class…….. She is not that bright” (CG17).*

#### Focusing attention

Clinicians specifically mentioned the term Attention Deficit Hyperactive Disorder (ADHD) as a specific outcome following CM. Parents did not mention this terminology.

*“Actually in their communities with children that have attention problems or become aggressive… they are being side-lined from school… the teacher is saying, “No we cannot manage your child, you need to go back.” In the village he is taken like a mental disturbed child” (Nurse 3)*

### General tasks and demands

#### Managing one’s own behaviour

Both families and professionals explained how behavioural problems and their manifestations had an impact on families and children and these were therefore a key outcome for families and children. Behaviour was described in a number of ways which included: aggression (beating up friends), being short tempered, “*change in personality”*, “*destructive behaviour*”, inappropriate behaviour and “undressing when playing”, *“behaving abnormally like a mad person” (amakhala ngati wamisala)” (CG5). This was also recognised by clinicians to be a big problem; “We are increasingly seeing this; a mother who says my child is absolutely fine they can walk but it’s not the child I had before they become very difficult, aggressive, can’t listen, they can’t do this, it’s not the same child I had before” (Clinician 4).*

### Communication

Speaking and communication were also frequently mentioned as an issue for children post CM. Parent/caregiversCG specifically mentioned the challenges with expressive language post CM; *“her limbs were dead and she could not speak. From then to now is not speaking” (CG14) “The problem I see at the moment is his speech, for him to speak clearly, to link words properly, it’s a challenge” (CG 8)*

### Mobility

Children with neurodevelopmental problems post CM were often described as having specific issues not just with movement but also with functioning and mobility. They were described as being reliant on parents/caregivers for activities of daily living and mobility. *“So this causes a challenge because quite often they are not feed themselves, they’re not able to support themselves, they are unable most of the times to walk and become completely dependent to the people who are around them. So for their quality of life to be achieved, it is dependent of the people who surround them” (Rehab/palliative care).*

### Respiratory function

Some professionals also described the risks of related chest infections as long-term sequelae of children affected by CM; *“When you arrive at the house and hear the breathing and you realize that the chest is congested because of the feeding practice. The big problem is the method of feeding, so you show them the correct position for feeding because if the child is being fed whilst lying down, the child is at risk of choking and also of pneumonia”* (*Rehab/*Palliative care).

### Self-care

Issues emerged often relating to the ability to feed, self-care and toilet. This linked to family ability depending on their efficacy and environment.

#### Ability to feed

Some carers described outcomes relating to feeding, including difficulty swallowing and poor appetite requiring forced feeding and nasogastric tube feeding; *“we were forcing him, opening his mouth and pouring in the porridge” (*CG7).

#### Toileting

For some, toileting was also an issue; “*when she wants to answer nature’s call I have to help her. If I am not around I would find she has faeces in her underwear and then I take care of it….. So my worry is she is growing and my strength is getting depleted.” (CG17)*

### Domestic life

Carers reported how children could and wanted to try and participate in simple tasks such as cooking nsima, but needed support to do so, as in the example below of a girl with hemiplegia; *“On the side of cooking, she could cook but not preparing nsima due to her arm condition but she is able to put cold water on fire she holds with one arm and also is able to set fire. When you see that she can’t manage you stop her and help.” (CG11)*. Some parents/caregivers with children with cerebral malaria, even noted an improvement in the child post illness. The description of improvement related not to an improvement in an impairment for the child but to their ability to participate in domestic life; *“On the house chores before she fell ill she had problems with carrying out her chores but when she fell ill there was an improvement” (CG13).* Other parents/caregivers similarly described an improvement through rehabilitation as the ability to do errands around the house; *“Sometimes when you are talking to him he would just be looking at you without doing anything. But since I started coming here he is able to do something when you send him on an errand. He is able to grasp it quickly and do it.”* (*CG6)*

### Interpersonal interactions

Parents/caregivers and professionals were greatly concerned about behavioural problems mainly in terms of the child’s interpersonal interactions with others; *“people I live with, some complain/worry…. the way she acts, just walking, running away like that makes people worry because by now she could have been in standard five, she could have been in school because I see her as being intelligent” (CG4).* Some carers even described social communication difficulties after CM; *“when she is with her friends, she isolates herself and plays in her own, she would be in one place and her friends would be elsewhere” (CG10).* Interpersonal difficulties were sometimes described as relating to the child’s physical disability or their ability to participate in play, or school; *“They cannot socialize with friends and the progress in school is not good as it was before he is like isolated because he cannot run, jump with friends and s/he cannot be in the same place with friends so the patient is also psychologically affected which also affect the academic progress” (Nurse 3).*

### Major life areas

Issues emerge regarding child education and the effects of a child post-CM on work and employment of parents and caregivers. Financial consequences were often highlighted. The primary CG, often the mother, was faced with challenges of meeting the needs of her child and finding time for demands of daily living; *“Most of the time I don’t go to the garden. I would just say this year I have not cultivated. I fail to go to the garden. I don’t have maize. I have to beg to get food” (CG10). “So when I am away may be to the market. When she wants to respond to nature’s call, it’s her friends who carry her. But they are growing and they also need to go to school.”CG16*

#### Financial consequences and competing priorities on the CG (economic life)

Parents/caregivers and health professionals cited the cost of managing a child with CM as a vitally important outcome for families. In addition to increased expenditure, carers discussed how there could be loss of business; *“if you are a business woman like me or you are working, you may quit your job to care for this child” (CG5) but also loss of revenue really affected them; “we had our own programs for people to see that this is a home. We planned to build a house. But we did not since the illness affected our savings when I was in the hospital” (CG9).*

### Community, social and civic life-implications for the child with CM and the family

Difficulties in balancing caring for a child with other responsibilities were mentioned by parents/caregivers and professionals. Health workers reported marriage breaking downs and mothers lamented leaving chores and responsibilities to children *“Sometimes you would see that the family would leave the child and go to other duties like farming and leave a child unattended to…it is pathetic because there is no care to a child…it looks like the family members…get tired in caring” (Rehab/Palliative care)*

Some parents/caregivers explained how they sought company of other women with similar problems to alleviate feelings of isolation; *“At* Cheshire Homes (centre for children with disabilities), *that is where I go when I feel that I need to be there, I go to Cheshire Homes…to learn how other children are doing, to learn how other parents are handling their children to learn how I can handle my child” (CG5).* Parents/caregivers expressed concerns about the future of affected children; *“The child is growing and I’m also getting old. ………So my concern is will the one who will take care of her take her like this? That’s my worry. It would have been better if she was able to walk so that when you send her to carry some chores she would do so. But the way she is would someone else be able to take care of her the way I am doing” (CG18).* Some parents/caregivers even mentioned children’s willingness to help fellow children; *“When her friends are seeing me carrying her, (they) would be asking me why she is not walking, I would say she is sick and they would say we will be helping her to walk.” (CG15)*

### C. Contextual and environmental influences

#### Environmental influences

The environment as well as societal attitudes obviously influences how parents/caregivers handled children. This was related to physical access and freedom within a setting where it was acknowledged children had disabilities; *“Whilst here in the hospital they had an access of going to a play room, they were chatting (with the child), but when they are at home they dump the child because of what the society are saying.” (Palliative/Rehabilitation team)*

#### Attitudes

Parents and professionals emphasised that community attitudes to children with disabilities and CM affect a child’s social life and ability to participate in daily activities. Palliative care members and parents/caregivers reported children being locked in the house in fear of ridicule from the community, or in order to free the parent to carry out other duties; *“Some are ashamed or embarrassed that people in the village will say that ‘at so and so’s house, there is a child like this.’ This is the first one, they are embarrassed. We could call it stigma right?” (Rehab/Palliative care).* Furthermore, behavioural problems in children with CM affected attitudes and inclusion; *“let’s say somebody has some, behavioural change. Usually they are a problem in the society. They can’t cope, I mean they can’t, ahh they can’t cope, they can’t play with friends all the time, they may be beating them up” (Rehab/Palliative care).*

The problem of stigma frequently arose with parents/caregivers who perceived it as a consequence of having a disabled child with CM. Often this was described as leading to feelings of isolation, loneliness and shame; *“My social life is really affected, when I go out, my neighbours shun me (amandisala)… I’m just alone in my house with my child… When I go outside, It is really a reproach (chimakhala ndithu chitonzo)” CG8*

### Services, systems and policies

#### Access to health-care

Some health workers perceived that children with neurodisabilities post-CM were treated differently with unnecessary referrals to tertiary care by local health workers who did not feel equipped to manage children with behavioural problems, adding to the burden for these families; *“If a child with a neurological problem suffers from malaria you would see that kind of attention this particular child will receive is completely different from the abled child…it is like is in already in a hopeless situation even with the knowledge that malaria is curable…in primary level in the health centre these children shall always end up being referred back to the referral hospital” (Clinician 2).* It was clear from the transcripts that there were few services, supports and policies to lessen the impact of having a child with a disability secondary to cerebral malaria. There was no mention of supportive schools, inclusion policies or supportive respite environments organised outside the home or immediate community.

## Discussion

The burden of CM is high but not just in terms of mortality but also in terms of the longer term consequences that it can inflict. More than 30% of survivors have sequelae, some which are more obvious than others but many which impact dramatically on the family.. Studies are no longer just looking at survival rates but are examining the long-term implications of neuro-infectious diseases such as CM. For this reason, there is need to expand the types of measured outcomes to also include functioning and participation of children post CM. Relevant outcomes relate to clinical and family care, including patient and carer’s perspectives which are essential to ensure key outcomes are assessed. This is the first qualitative study of opinions of parents/caregivers and health professionals exploring perceptions of CM outcomes of children with and without neurodisabling sequelae. Growing evidence from studies in Africa demonstrated relevance to the short and long-term outcomes of CM [10]. Most reports concentrated on assessment of functional impairments, without attention to socialisation, schooling and participation. This study has demonstrated that parents/caregivers as well as professionals who work with these families place importance both on acquired impairments as well as on the socialisation of children, their participation within society, and their functioning and financial situation. This emphasis and related qualitative outcomes are likely to be similar for other persisting and debilitating neurodisabilities such as encephalitis, tuberculous meningitis, paediatric HIV and neurocystercercosis.

For families and professionals in Malawi, the impact of CM goes beyond the individual to the wider effect on the family, which is influenced by the environment and social context [15].

Disability causes a large burden of disease within settings such as Malawi, [32]. Other studies, in settings similar to Malawi, have clearly shown how it is not just the physical impact of having a disability which can lead to problems for children and families, but that the strong cultural beliefs and stigma attached to disability can have a huge impact on individuals and families, and influence their response and approach to child care [33, 34]. Certain conditions such as epilepsy/seizures are often associated with witchcraft and cause stigma and discrimination for the family [33, 35]. The aetiology of some conditions such as epilepsy and club foot have been considered contagious, sometimes stigmatizing families even further [34, 35].

Outcomes relating to neurological structure and function such as neurological impairment and epilepsy are regularly described in the literature and more recently there has been a growing interest in the cognitive outcomes of CM, but the social implications of having a child with a disability have not been adequately considered in outcome studies. In previous qualitative studies Malawi, themes of isolation, dependence, limited participation in school, play and other spheres of life were realities for children and families with disabilities (musculoskeletal impairments [32, 33]. Disabled children were described as being “left behind” (locked in the house or lagging behind in school), and “left out” (of activities of the home and social life) [32]. Although children were not interviewed, the present findings were similar to those of studies, which included child interviews [36]. The family stresses such as financial and time constraints, and the emotional impact of worry and frustrations, have also previously been described [37].

We explored outcomes of interest to parents, caregivers and health professionals relating to CM survivors. This is one step in the process of developing a COS [19]. Next steps involve exploring existing knowledge through reviews of the literature, which could then be mapped more systematically on to an ICD framework. A consensus process would then enable a final outcome score list to be generated. We hope that by using this framework we would be able to recommend a series of outcome measures that could either be used as they are or may need to be adapted and validated for use in populations such as this one in Malawi in the future. To undertake this work we would aim to continue to incorporate the opinions of key players (experts, families, parents, caregivers and patients). This approach has recently been undertaken successfully in the UK [38, 39]. This paper highlights the value of including patients and the public in the process, particular in a developing country setting where this has been rarely done.

Consultative processes conducted for COS in areas of rheumatology, asthma and fibromyalgia have generated new outcomes and have required new assessment measures to be considered or developed [19, 40, 41]. In view of the findings of this study, a similar process may be required in order to include the priorities identified by the stakeholders, in particular, measures that assess participation of children within home and school situations, as well as measures assessing family stress.

### Study Limitations

Some of the study participants were children in a CM follow-up study and the responses of the participants, may have been influenced by this participation. Recruitment was broader than this cohort and included other settings, local support groups, and other rehabilitation clinics. In this study, 19 carers were interviewed through purposive sampling. There was a mix of carers of children from different ages, from rural/urban settings and of those with or without persisting neurodisabling sequelae of varying severity and it is difficult to determine the extent of selection bias. This may have limited response categories. Some health professionals may have been influenced by previous work in outcome studies with CM children. Several health professionals interviewed worked in other hospital and community areas, which should reduce selection bias. The use of multiple qualitative data collection methods such as focus group discussions could improve triangulation, as this would allow participants to reflect on and clarify their thoughts and to share ideas. Analysis through NVIVO did enable mapping on to the ICF CY constructs although this was not done in a quantitative fashion. Additional measurement of the number of quotes/themes derived in each construct would be useful, but require a larger sample size. Reliance on participants from around Southern Malawi alone may limit regional representativeness.

## Conclusions

Findings encompassed all aspects of the ICF framework and clearly demonstrated perceptions of the far-reaching consequences of CM on individuals, families and the wider community. For a COS to be useful and relevant to the needs of the participants, it must take account of the range of outcomes described including quality of life and community participation and incorporate them into a consensus process.

## Additional files

Additional file 1: Topic guide for Interviews for health professionals. (DOCX 12 kb)
Additional file 2: topic guide for Interviews for caregivers. (DOCX 14 kb)

## Abbreviations

CG: Caregiver
CM: Cerebral Malaria
COS: Core Outcome Sets
ICF: International Classification for Functioning and Disability
WHO: World Health Organisation
