# Supplementary material for: Exploring neurodevelopmental outcome measures used in children with cerebral malaria: the perspectives of caregivers and health workers in Malawi
Source: BMC Pediatr. 2017 Jan 10;17:9. doi: 10.1186/s12887-016-0763-y (PMC5223588; doi:10.1186/s12887-016-0763-y)
Supplement: Additional file 1: — Topic guide for Interviews for health professionals. (DOCX 12 kb) [file 12887_2016_763_MOESM1_ESM.docx]

**Supplementary file 1: topic guide for Interviews for health professionals**

Aim: To explore health professionals’ views on the neurodevelopmental assessment tools currently in use and what outcomes they think are important to test in children who have had cerebral malaria

This is simply a guide and is flexible. The questions do not have to be covered in the listed order but should be discussed.

**Opening:**

Once informed consent has been obtained, welcome participants, encourage them to feel free and be assured of confidentiality

**Discussion**

Tell me about how are you involved in the care of children with cerebral malaria..

Tell me about the range of problems you see in these children?

Tell me about the problems you think caregivers often site as concerns in these children?

What do you think they hope will get better in their children?

What are they worried might happen to their children after they have been unwell?

What do you think is the most important?

What do you think should be the focus when looking at outcomes of children with cerebral malaria?

What has been your experience with tests for neurological disability and outcomes so far?

From your experience what outcomes do you think are important to be measured?

Can you rank them in order of importance from the most important to the least important?

Do you have any questions?

Thank them for their involvement and assure them of confidentiality and that what has come up in the discussion should discuss elsewhere
