# Supplementary material for: Exploring neurodevelopmental outcome measures used in children with cerebral malaria: the perspectives of caregivers and health workers in Malawi
Source: BMC Pediatr. 2017 Jan 10;17:9. doi: 10.1186/s12887-016-0763-y (PMC5223588; doi:10.1186/s12887-016-0763-y)
Supplement: Additional file 2: — topic guide for Interviews for caregivers. (DOCX 14 kb) [file 12887_2016_763_MOESM2_ESM.docx]

**File 2: Supplementary file showing topic guide 2: Interviews for caregivers**

**Aim** To explore caregivers’ views on which neurodevelopmental outcomes they consider important to investigate in children who have had cerebral malaria

This is simply a guide and is flexible, it will be modified and refined during the research process as data is collected. The questions do not have to be covered in the listed order but should be discussed.

**Introduction**

Explanation about the project and its aims

**Opening**

Once informed consent has been obtained, welcome participants, encourage them to feel free and be assured of confidentiality. Explain the use of the Dictaphone and ask permission for its use. Remind them that they do not have to answer any question they do not want to.

This is simply a guide and is flexible. The questions do not have to be covered in the listed order but should be discussed.

**Topic guide:**

Experiences since the child had cerebral malaria

Tell me how things have been since your child had cerebral malaria?

Kodi mwana wanu wakhala ali bwanji chidwalileni malungo akulu?

What do you feel is going well or improving?

Zome mukuwona kuti zakuyenda bwino ndi chani?

Tell me….What has changed since your child had cerebral malaria?

Chomwe chasintha ndi chani mwana wanu chidwalileni malungo akulu?

What differences if any do you see between how the child was before the disease and now?

Mukusiyanitsa bwanji pakati pa mmene mwana wanu anali asanadwal malungo akulu ndi pano?

Tell me more about this…..

What concerns you about the child’s condition at the moment?

Kodi chomwe chimakudetsani nkhawa mmene mwana alili panthawi yino ndichani?

What can be the effect of having had cerebral malaria

Kodi malungo akulu atha kukudza bwanji banja kapena mwana yemwe anadwalapo malungo akulu?

Is the child attending school? If so, how is this going?

Kodi mwana ali pa sukulu? Ngati ali, zikuyenda bwanji?

Knowledge about the child’s condition

What do you understand about your child’s condition?

Kodi chomwe mumdziwa zokudza mmene mwana alili pakali pano ndi chani?

What would you like to know about your child’s condition?

Kodi mungafune kudziwa chani pa izi?

What kind of things do you think it is important to know?

Ndi chani chomwe mukuwona ngati ndichofunikira kudziwa?

Service/treatment priorities

Tell me about the things your child needs help with?

Ndi chani chomwe mwana wanu amafuna chithandizo chapadela pochita?

Tell me about the biggest problems for you at the moment?

Kodi bvuto lomwe mukukumana nalo panthawi yino ndiyanji?

Tell me about what you have discussed about your child’s condition with your family/ with people outside your family?

Mwakambilana chani zokudza mwana wanu ndi achibale kapena anthu akunja kwa banja lanu

Tell me about the condition of your child now?

Anthu ena amaganiza chani za mwana wanu?

Can you tell me….What do you think are the important things that children who survive cerebral malaria might need?

Inu mukuganiza kuti zinthu zofunikira kuthana nazo mwa mwana wodwala malungo akulu ndi chani?

Debriefing for participant

At the end – Summary of discussion will made be made and participant will be asked to comment or add.

Check if they have any concerns or questions

Thank them for their involvement and assure them of confidentiality;

Document findings in diary and with research assistant on impressions;

Reflect on what went well/badly, what should be changed for next time and what main issues that came out of the interview.

Complete note writing.

Download conversation from Dictaphone and save and backup conversation.

Keep all front sheets with patient demographics in site file.
